# Supplementary material for: Need for cognition moderates the relief of avoiding cognitive effort
Source: PLoS One. 2023 Nov 16;18(11):e0287954. doi: 10.1371/journal.pone.0287954 (PMC10653461; doi:10.1371/journal.pone.0287954)
Supplement: S1 File — (PDF) [file pone.0287954.s001.pdf]

Contents

Supplementary Results 2

Experiment 1 2

Experiment 2 6

Supplementary Results

Experiment 1

Comparison of difficulty level and arithmetic accuracy in predicting hedonic responses.

Our main results showed that hedonic responses to the gambling outcome (measured through reward-related feedback ratings) were best explained by a model that contained the *outcome x difficulty level* interaction. An outstanding question pertains whether the factor difficulty level, or rather the individual accuracy within each difficulty condition, better predicts the feedback ratings. To address this question, first we compared two models including either the *outcome x difficulty level* or *outcome x accuracy*. In the latter model, accuracy is a continuous predictor corresponding to average accuracy for each participant and each difficulty level. We found that data under a model including *outcome x accuracy* were BF = 1.84×10^{3} more likely than under a model including *outcome x difficulty level*.

Second, we compared iterative models that included either a single interaction (*outcome* x *accuracy*) or both interactions (*outcome* x *accuracy* + *outcome* x *difficulty level*). Note that with this syntax we implied the specification of the main effects of the interaction terms. This comparison allowed us to assess whether adding the interaction of *difficulty level* with *outcome* improved the model over and above the interaction of *accuracy* with *outcome* alone (i.e., we controlled for the variance explained by this latter interaction). We found that the data under the model including both interaction terms was BF = 95.57 times less likely than under a simpler mode including the interaction of *accuracy* with *outcome* alone. Conversely, the model including both interaction terms performed better than a simpler model including the interaction of *difficulty level* and *outcome* alone (BF = 19.27).

Together, these analyses suggest that the interaction including *difficulty level* does not have explanatory power above the one including *accuracy.*

Interactive effect of outcome with difficulty level when predicting 'relief' ratings alone

In the main analyses of gambling task feedback ratings, we predicted the VAS % rating of all the three affective dimensions, to estimate the underlying hedonic response. Here, to validate the choice of 'relief' to describe the interaction of the factors *outcome* and *difficulty level*, we rerun the same analysis, but we filter the data to predict selectively the 'relief' affective dimension. Similar to the main analysis, the models of interest included the effects of 1) outcome, 2) difficulty level, 3) outcome + difficulty level, and 4) outcome x difficulty level. The factor Subject was treated as nuisance.

While in the main analysis the 'relief' ratings of the gambling feedback were best explained by a model that contained the *outcome* x *difficulty* level interaction, here that model was performing similarly to a simpler model including the main effect *outcome*. Under the interaction model the observed data were BF10 = 1.65×10^{201} times more likely to be produced than under the null model. Under the simpler *outcome* model the observed data were BF10 = 2.73×10^{201} times more likely to be produced than under the null model. The two models respectively explained the observed data 11 and 19 times better than the third-best model, which included the two main effects of *outcome* and *difficulty level*.

Subjective ratings across the three affective dimensions are correlated

We observed very robust correlations between pairs of affective dimensions. These correlations were calculated within participant, across outcome and difficulty conditions. Supplementary Table 1 provides the group-level statistics, including the mean and standard deviation of these correlations.

	Dimensions	Mean	SD
1	pleasure & relief	0.85	0.14
2	pleasure & 1-frustration	0.81	0.12
3	relief & 1-frustration	0.71	0.18

Supplementary Table 1

Participant showed internal consistency in reward-related feedback ratings

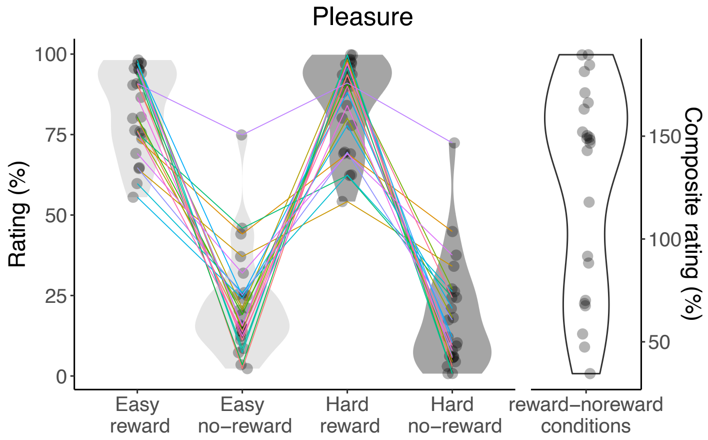

Supplementary Figure 1

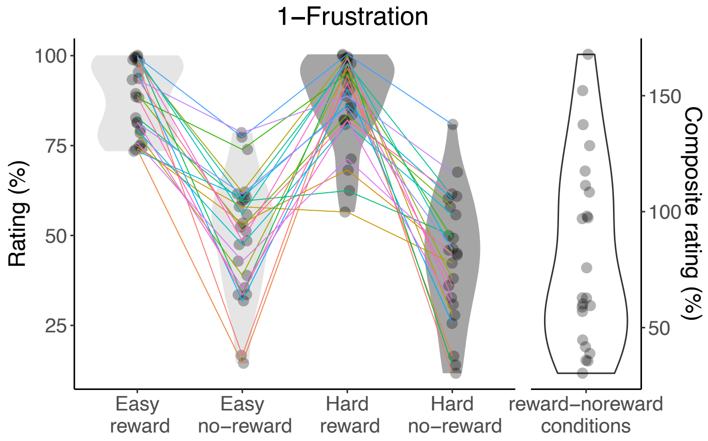

Supplementary Figure 2

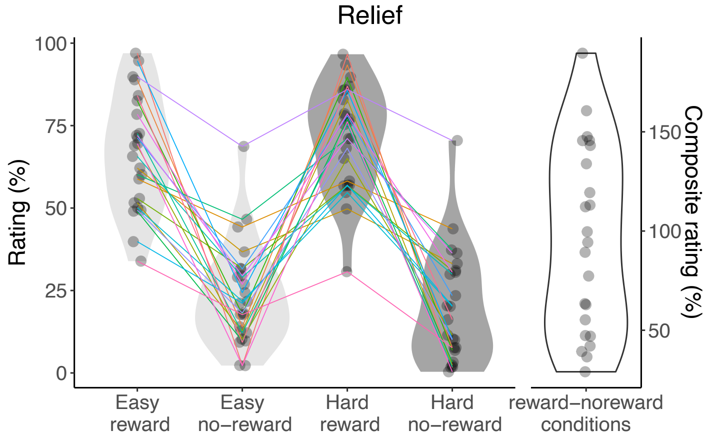

Supplementary Figure 3

Supplementary Figures 1-3 recapitulate the results in the main Figure 2, but each of them now refers to a single affective dimension, and outcome levels (reward or no-reward) for each difficulty level are adjacent. Participant's datapoints (mean VAS %) are connected with colored segments to highlight the pattern of ratings across conditions. The secondary plot on the right shows a composite rating, obtained by summing ratings from reward conditions and subtracting the ones from no-reward conditions. We observe an overall internal consistency across outcome levels (i.e., lower values for no-reward vs reward). Some participants showed less variance than others in their responses across conditions, demonstrating a preference for a narrow section of the VAS scale (i.e., showing high precision in their ratings across conditions).

Experiment 2

Comparison of difficulty level and arithmetic accuracy in predicting hedonic responses.

In analogy to Experiment 1, here we first compared two models including either the *outcome* x *difficulty level* or *outcome* x *accuracy.* In the latter model, accuracy is a continuous predictor corresponding to average accuracy for each participant and each difficulty level.

At odds with Experiment 1, we found that data under a model including *outcome x accuracy* were BF = 4.71 x 10^{14} times *less* likely than under a model including *outcome* x *difficulty level*.

Second, we compared iterative models that included either a single interaction (*outcome* x *accuracy*) or both interactions (*outcome* x *accuracy* + *outcome* x *difficulty level*). Note that with this syntax we implied the specification of the main effects of the interaction terms. This comparison allowed us to assess whether adding the interaction of *difficulty level* with *outcome* improved the model over and above the interaction of *accuracy* with *outcome* alone (i.e., we controlled for the variance explained by the latter interaction). At odds with Experiment 1, we found that the data under the model including both interaction terms was BF = 3.01 x 10^{14} times more likely than under a simpler mode including the interaction of *accuracy* with *outcome* alone. Conversely, the model including both interaction terms did not perform better than a simpler model including the interaction of *difficulty level* and *outcome* alone (BF = 0.63).

Together, these analyses suggest that the interaction including *difficulty level* has strong explanatory power over and above the one including *accuracy.* In other words, the interaction of *outcome* with *difficulty level* survived after controlling for the interaction of *outcome* with *accuracy*.

Interactive effect of outcome with difficulty level when predicting 'relief' ratings alone

In the main analyses of gambling task feedback ratings, we predicted the VAS % rating of all the three affective dimensions, to estimate the underlying hedonic response. Here, to validate the choice of 'relief' as construct to describe the interaction of the factors *outcome* and *difficulty level*, we rerun the same analysis, but we filter the data to predict selectively the 'relief' affective dimension. Similar to the main analysis, we compared models including the effects of 1) outcome, 2) difficulty level, 3) outcome + difficulty level, and 4) outcome x difficulty level. The factor Subject was treated as nuisance.

In line with the main analysis, the 'relief' ratings of the gambling feedback were best explained by a model that included the *outcome* x *difficulty* level interaction. Under this model the observed data were BF10 = 1.31 x 10^{398} times more likely to be produced than under the null model. Moreover, the outcome x difficulty model explained the observed data 2.49 x 10^{5} times better than the second-best model, which only included the main effect of outcome.
Additionally, under a model 5) including the three-way interaction of *outcome* x *difficulty level* x *NFC*, the data were more likely compared to the former best model (BF = 24) and the null model (BF10 = 3.27 x 10^{399}).

The moderating effect of NFC persists after controlling for arithmetic performance

We argued that NFC moderated the interactive effect between *outcome* and *difficulty level*, i.e. the "relief effect" of avoiding higher vs. lower anticipated effort. Yet, in light of the fact that participants scoring high on NFC also solved the hard arithmetic task faster and better than those low on this dimension, we also warned caution when interpreting the modulatory influence of the NFC on the relief effect. In fact, changes in the 'relief' effect may be driven by differences in intrinsic motivation to engage with effort, as captured by the NFC questionnaire, but perhaps also

by lower objective difficulty for participants scoring low in NFC. Further, another possibility is that participants scoring low in NFC, and performing sub-par in the arithmetic task, developed lower expected values for high difficulty trials, corresponding to lower estimates of reward probability. Therefore, the relief for avoiding high effort may be compounded by the relief for avoiding risky trials (i.e., risk or loss aversion).

Therefore, we run an additional control analysis where we assessed the moderating role of NFC on the relief effect while controlling for individual performance. We defined a new continuous predictor, "delta_performance", corresponding to the difference in mean accuracy between the hard and easy arithmetic difficulty levels, computed for each participant. Next, we added this predictor in a new model including the three-way interaction *outcome*effort*delta_accuracy*. We reasoned that the larger *delta_performance*, the larger the difference in expected reward between conditions. We expected this interaction to compete for some of the variance explained by the three-way interaction *outcome*effort*NFC.* Crucially, we also predicted that a model including the interactions with *delta_accuracy* (*outcome*effort*delta_accuracy*) *and* the interactions with *NFC* (*outcome*effort*NFC*) would explain the data better than one including the interactions with *delta_accuracy* alone.

As predicted, the data were 6.93×10^9 times more likely under the more complex model, including both *outcome*difficulty level*NFC* and *outcome*difficulty level*delta_accuracy*, compared to a simpler model including only *outcome*difficulty level*delta_accuracy.*

When comparing the moderating roles of *delta_accuracy* and *NFC*, the three-way interaction model including NFC performed much better than the three-way interaction model including delta_accuracy (see Supplementary table 2, which summarizes these comparisons). Taken alone, the model including the three-way interaction of *outcome*, *difficulty level*, and *delta_accuracy* performed around 57 times better than the simpler model including the two-way interaction between *outcome* and *difficulty level,* suggesting some role of arithmetic performance in affecting the ratings, and the relief effect in particular. Altogether, these results suggest that the individual arithmetic ability may play a role in the hedonic responses to effort avoidance, possibly through changes in expected reward between difficulty conditions, and therefore determining a variable impact of loss aversion. Importantly, this result also indicates that these alternative hypotheses do not rule out the complementary and stronger role of individual disposition towards effortful tasks (NFC) in moderating the relief effect.

Model	BF10	% pe
[1] Subject * AffDim + outcome * difflev * NFC + outcome * difflev * delta_acc	6.93 x 10^9	±27.31%
[2] Subject * AffDim + outcome * difflev * NFC	3.55 x 10^10	±16.18%
Against denominator		
percent ~ Subject * AffDim + outcome * difflev * delta_acc		

Supplementary table 2

Subjective ratings across the three affective dimensions are correlated

We observed very robust correlations between pairs of affective dimensions. These correlations were calculated within participant, across outcome and difficulty conditions. Supplementary Table 3 provides the group-level statistics, including the mean and standard deviation of these correlations.

	Dimensions	Mean	SD
1	pleasure & relief	0.80	0.19
2	pleasure & 1-frustration	0.67	0.32
3	relief & 1-frustration	0.57	0.37

Supplementary Table 3

Participant showed internal consistency in reward-related feedback ratings

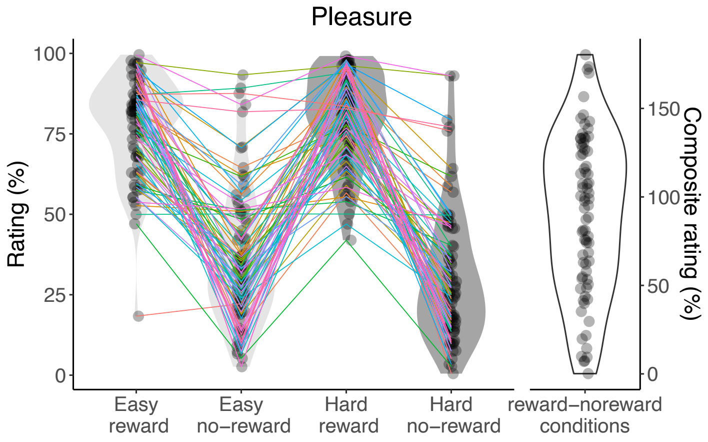

Supplementary Figure 4

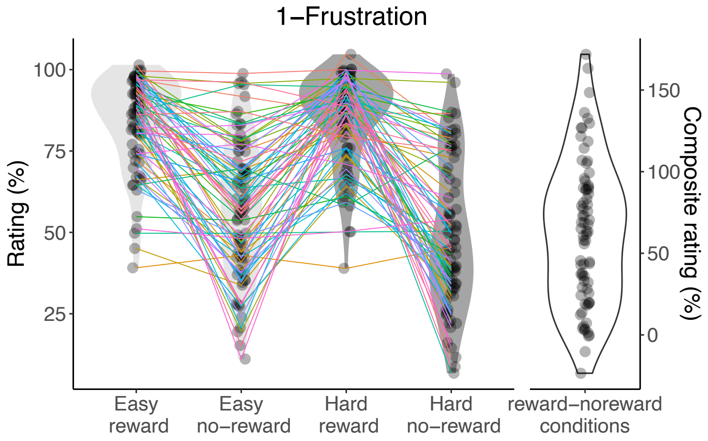

Supplementary Figure 5

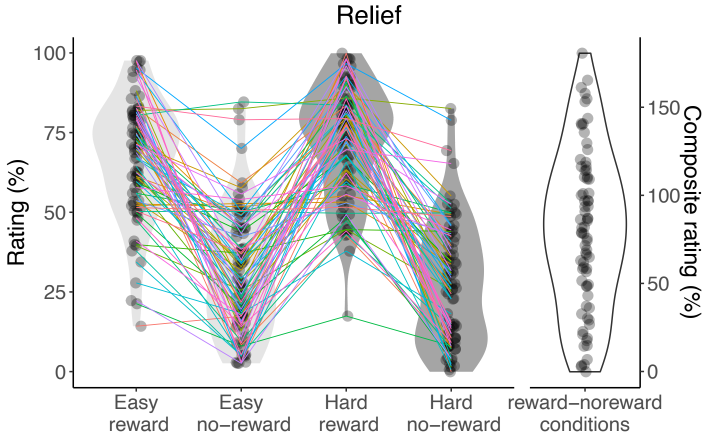

Supplementary Figure 6

Supplementary figures 4-6 recapitulate the results in the main Figure 3, but each of them now refers to a single affective dimension, and outcome levels (reward or no-reward) for each difficulty level are adjacent. Participant's datapoints (mean VAS %) are connected with colored segments to highlight the pattern of ratings across conditions. The secondary plot on the right shows a composite rating, obtained by summing ratings from reward conditions and subtracting the ones from no-reward conditions. We observe an overall internal consistency across outcome levels (i.e., lower values for no-reward vs reward).
This is especially the case for the affective dimensions "relief" and "pleasure", while a few participants showed a modest reversal of this pattern for the dimension "frustration" (see negative values in the composite rating).
Some participants showed less variance than others in their responses across conditions, demonstrating a preference for a narrow section of the VAS scale (i.e., showing high precision in their ratings across conditions).
