# Supplementary material for: Need for cognition moderates the relief of avoiding cognitive effort
Source: PLoS One. 2023 Nov 16;18(11):e0287954. doi: 10.1371/journal.pone.0287954 (PMC10653461; doi:10.1371/journal.pone.0287954)
Supplement: Attachment — Submitted filename: Response to Reviewers.pdf [file pone.0287954.s003.pdf]

PONE-D-23-18706
Need for cognition moderates the relief of avoiding cognitive effort
PLOS ONE

Reviewer 1

> I appreciate that the authors saw my concerns here and agreed this is worthy of addressing. The analysis which was run however isn't exactly what I had in mind, which may be due to a lack of clarity on my part. The more appropriate analysis would compare iterative models —rating ~ outcome vs. rating ~ outcome + accuracy vs. rating ~ outcome + accuracy + difficulty. The crucial step here is comparing the last two models as it allows you to assess whether adding difficulty improves the model fit above accuracy alone. This is roughly equivalent to what Westbrook et al. (2013) + others have done.
>
> While I don't think not doing this precludes publication, I think the authors should consider this approach and include it in the main manuscript (or at least supporting information) if they agree. The current approach in the Supporting Information is OK, but it doesn't allow you to assess whether difficulty adds anything over and above accuracy. The authors, however, are of course correct in saying the current experiment doesn't allow this issue to be resolved entirely and it's a good suggestion to note this in the General Discussion, as they have.
>
> I believe all the other amendments to both mine and Reviewer 2's suggestions are appropriate and have hopefully improved the paper!
> Good work again on an interesting pair of experiments. I've enjoyed reading this work.

We thank the reviewer for his positive evaluation of our revised manuscript, and for wanting to further clarify how to best approach this analysis. Indeed, comparing iterative models including *additional* predictors, keeping every other predictor equal, should allow to verify the existence of additional explanatory power of the added predictors.

We implemented the suggestion of the reviewer, but rather than adding the simple main effects of outcome, accuracy, and difficulty iteratively, we decided to compare a model including the main effects *and* interaction of outcome and accuracy, to a model including those same predictors *and* the interaction of outcome and difficulty level. We believe that this approach does capture the iterative logic suggested by the reviewer (and implemented in Westbrook et al., 2013), but is better suited to answer our research question. In fact, the main effects of *difficulty level* and *accuracy* alone do not explain much variance in the data, but they do show strong interactions with *outcome*.

Our results confirm the pattern of results previously reported in the Supplementary Information. For Experiment 1, a simple model including the interaction of *accuracy* with *outcome* alone performed better than the model including both interaction terms,

suggesting the interaction with *difficulty level* does not have explanatory power above the interaction of *outcome* with *accuracy*. Rather, the more complex model with both interaction terms performed better than one including the simple interaction with difficulty level. For Experiment 2, the opposite pattern was found, in line with the analysis reported in the previous round of revision. More specifically, the model including two interaction terms performed much better than the one including the interaction of *outcome* with *accuracy* alone. In other words, the interaction of *outcome* with *difficulty level* survived after controlling for the interaction of *outcome* with *accuracy*. We have included these results in the Supplementary Information.

We are grateful to the reviewer for pointing out this additional approach which further clarifies that, in the second experiment, there is no evidence that individual differences in arithmetic performance (and therefore, in reward expectations) confounded the relief effect of avoiding high vs. low effort.

Reviewer 2

> I thank the authors for thoroughly addressing all of my comments. I have no further concerns and I am happy to recommend this manuscript for publication.

We are very grateful to the reviewer for the insightful suggestions offered in the previous round of revision, that strongly helped us improving the paper.
